# Targeted maximum likelihood estimation for a binary treatment: A tutorial

**DOI:** 10.1002/sim.7628

**Published:** 2018-04-23

**Authors:** Miguel Angel Luque‐Fernandez, Michael Schomaker, Bernard Rachet, Mireille E. Schnitzer

**Affiliations:** ^1^ Cancer Survival Group, Department of Non‐Communicable Disease Epidemiology Faculty of Epidemiology and Population Health, London School of Hygiene and Tropical Medicine London UK; ^2^ Department of Epidemiology Harvard T.H. Chan School of Public Health Boston MA USA; ^3^ Biomedical Research Institute of Granada, Non‐Communicable and Cancer Epidemiology Group (ibs.Granada) Andalusian School of Public Health Granada Spain; ^4^ School of Public Health and Family Medicine, Center for Infectious Disease Epidemiology and Research The University of Cape Town Cape Town South Africa; ^5^ Faculté de pharmacie Université de Montréal Montréal Canada

**Keywords:** causal inference, ensemble Learning, machine learning, observational studies, targeted maximum likelihood estimation

## Abstract

When estimating the average effect of a binary treatment (or exposure) on an outcome, methods that incorporate propensity scores, the G‐formula, or targeted maximum likelihood estimation (TMLE) are preferred over naïve regression approaches, which are biased under misspecification of a parametric outcome model. In contrast propensity score methods require the correct specification of an exposure model. Double‐robust methods only require correct specification of either the outcome or the exposure model. Targeted maximum likelihood estimation is a semiparametric double‐robust method that improves the chances of correct model specification by allowing for flexible estimation using (nonparametric) machine‐learning methods. It therefore requires weaker assumptions than its competitors. We provide a step‐by‐step guided implementation of TMLE and illustrate it in a realistic scenario based on cancer epidemiology where assumptions about correct model specification and positivity (ie, when a study participant had 0 probability of receiving the treatment) are nearly violated. This article provides a concise and reproducible educational introduction to TMLE for a binary outcome and exposure. The reader should gain sufficient understanding of TMLE from this introductory tutorial to be able to apply the method in practice. Extensive R‐code is provided in easy‐to‐read boxes throughout the article for replicability. Stata users will find a testing implementation of TMLE and additional material in the Appendix S1 and at the following GitHub repository: https://github.com/migariane/SIM-TMLE-tutorial

## 1 INTRODUCTION

During the last 30 years, modern epidemiology has been able to identify significant limitations of classic epidemiologic methods when the focus is to explain the effect of a risk factor on a disease or outcome.^1^, ^2^ In observational studies, treatment groups are typically not directly comparable; thus, a careful statistical adjustment for confounders is necessary to obtain unbiased exposure (or treatment) effect estimates. Failure to account for confounding variables, namely, those preexposure variables associated with both the exposure and the outcome, can result in a biased estimate.^3^ Causal inference based on the Neyman‐Rubin potential outcome framework^4^ allows researchers to carefully adjust for confounders under structural causal assumptions. The framework was first introduced in the social sciences by Rubin^5^ and later in epidemiology and biostatistics by Greenland and Robins.^6^

Causal effects are often formulated in potential outcomes, as formalised by Rubin.^4^ Let A denote a binary exposure, **W** a preexposure vector of potential confounders, and Y a binary outcome. Each individual has a pair of potential outcomes: the outcome they would have received had they been exposed (A = 1), denoted Y(1), and the outcome had they been unexposed, Y(0). However, it is only possible to observe a single realisation of the outcome for an individual. We observe Y(1) only for those in the exposure group and Y(0) only for those in the unexposed group.^4^, ^5^ Common causal estimands of interest are the average treatment effect (ATE), defined as E[Y(1) − Y(0)], and the marginal odds ratio (MOR), defined as E[Y(1)] × {1 − E[Y(0)]}/{E(1 − E[Y(1)]) × E[Y(0)]}.

To identify the ATE, classical epidemiologic methods, such as standard regression models where the treatment is included as a covariate in the analysis, require the assumption that the effect measure of the treatment of interest is constant across the levels of confounders included in the model.^7^ However, in observational studies, this is often not the case. In 1986, a seminal paper^6^ demonstrated that under untestable causal assumptions (conditional exchangeability, positivity, consistency, and noninterference), a consistent estimate of the ATE can be obtained by using the G‐formula^6^ (a generalisation of standardisation with respect to the confounder distribution):

$$\mathrm{ATE}=\sum_{W}\left[\sum_{y}P\left(Y=y|A=1,W=w\right)\;-\;\sum_{y}P\left(Y=y|A=0,W=w\right)\right]P\left(W=w\right)$$

G‐computation,^8^ based on the estimation of the components in the G‐formula, allows for a treatment effect that may vary across the levels of the confounders. This approach relies on parametric modelling assumptions and bootstrap for the estimation of the standard error. Therefore, G‐computation is sensitive to model misspecification^9^, ^10^ and requires time‐consuming estimation of confidence intervals (CIs).

Alternatively, the propensity score^11^ can also be used for estimation of the ATE. Propensity score methods, introduced by Rosenbaum and Rubin,^12^ estimate the treatment mechanism. In our setting, where treatment is assigned at a single time point, the propensity score is defined as the probability of being treated given the observed confounders **W**, denoted P(A = 1|**W**). The propensity score is used to statistically balance exposure groups in their preexposure covariates to estimate the ATE. This may be done via matching, weighting, or stratification.^13^, ^14^ When weighting by the inverse of the propensity score, extreme values of the propensity score can lead to large weights, resulting in unstable ATE estimates with high variance (in particular, ATE estimates can fall outside the constraints of the statistical model). Furthermore, when analysing observational data with a large number of variables and potentially complex relationships among them, model misspecification is of particular concern in this case as well.^10^ Hence, correct model specification is crucial to obtain unbiased estimates of the true ATE.^10^, ^14^, ^15^ Overall, the above‐mentioned methods can be classified as those focused on modelling the outcome‐generating function of treatment and confounders (ie, G‐computation) and those focused on modelling the treatment‐generating function of confounders (ie, propensity‐score methods).

Double‐robust methods were developed to minimise the impact of model misspecification.^10^, ^16^, ^17^ Double‐robust methods require estimation of both the outcome and treatment mechanisms. For the estimation of the ATE in our example, the outcome mechanism is the conditional expectation of the outcome given the exposure and the covariates, denoted E(Y|A,**W**), and the treatment mechanism corresponds to the propensity score, which is the conditional probability of being treated given the observed confounders **W**, denoted P(A = 1|**W**). Double‐robust means that the estimator is consistent as long as either the outcome model or the treatment model is estimated consistently. Some double‐robust estimators are also locally semiparametric efficient, meaning that the estimator has minimal large sample variance among estimators that make the same model assumptions, under the correct specification of the required models.^10^, ^16^, ^18^

Current simulation‐based evidence shows that the use of double‐robust and locally efficient methods such as the augmented inverse probability of treatment weighted (AIPTW) and the targeted maximum likelihood estimation (TMLE) method estimators often outperform the G‐computation and propensity score methods, in both point and interval estimation.^10^, ^16^, ^19^ However, AIPTW is less robust to data sparsity and near violations of the practical positivity assumption than TMLE (ie, when certain subgroups in a sample rarely receive some treatment of interest).^10^, ^16^, ^19^

Targeted maximum likelihood estimation, a general template for the construction of efficient and double‐robust substitution estimators, was first introduced by Van der Laan and Rubin in 2006^20^ but is based on existing methods.^18^, ^21^ This approach first requires a specification of the statistical model, corresponding with what restrictions are being placed on the data‐generating distribution. For the ATE, the TMLE procedure requires initial estimates of E(Y|A,**W**) and P(A = 1|**W**), and then includes a substitution “targeting” step that optimises the bias‐variance tradeoff for the targeted parameter (e.g., the ATE). Furthermore, one can readily use ensemble and machine‐learning algorithms to estimate E(Y|A,**W**) and P(A = 1|**W**), thus avoiding model misspecification.^10^

Targeted maximum likelihood estimation respects the limits of the possible range of the targeted parameter, reduces bias through the use of ensemble and machine‐learning algorithms, and, finally, makes statistical inference quick and easy based on the efficient influence curve (IC).^19^, ^20^, ^22^, ^23^ Evidence shows that TMLE can provide consistent ATE estimates in challenging settings and has a smaller asymptotic variance compared with other (nonefficient) double‐robust estimators.^10^, ^19^, ^24^ Mathematically, TMLE and AIPTW are both efficient and have the same asymptotic properties. In particular, they both have the minimum asymptotic variance in their class of semiparametric estimators. However, TMLE is a substitution estimator and behaves differently in finite sample settings producing estimates in the range of values of the parameter space while AIPTW does not.^10^, ^19^, ^24^

The statistical properties of TMLE make it a suitable tool for applied researchers aiming to estimate causal effects. The TMLE procedure is available in standard statistical software such as the ***tmle*** package^19^ implemented in the statistical software R (R Foundation for Statistical Computing, Vienna, Austria). Users familiar with other software can also easily use TMLE, for example, by using our test version for a TMLE implementation in Stata or simply by importing their data to R (see Appendix S1).

We provide a step‐by‐step guided implementation of TMLE by using a simulated example inspired by population‐based cancer research.^25^ We aim to demystify TMLE and show applied medical statisticians how they can easily adopt the method for their applications. In contrast to many other papers, we demonstrate the implementation of TMLE in a realistic setting with many challenges such as near‐positivity violations (ie, manifested by large inverse probability of treatment weights) and misspecification of both the treatment and outcome models. We also show the implementation of AIPTW and the benefit of incorporating advanced machine‐learning algorithms to reduce bias. We provide commented R code embedded in boxes as well as Stata code in the Appendix S1, making our results fully reproducible.

## 2 MOTIVATIONAL EXAMPLE, CAUSAL MODEL, AND DATA GENERATION

The motivating example was developed to estimate the 1‐year mortality risk difference and odds ratio of death (Y) for cancer patients treated with monotherapy (only radiotherapy; A = 1) versus dual therapy (treatment with radiotherapy and chemotherapy; A = 0) while controlling for possible confounders (**W**).^25^, ^26^ To be able to consistently estimate the ATE, the data must satisfy the following assumptions: (i) Cancer treatment is independent of the potential mortality outcomes after conditioning on **W** (ie, (Y(0), Y(1))⊥A|**W**). This assumption is often referred to as “conditional exchangeability” and one cannot test it using the observed data. It implies that (within the strata of **W**) the mortality risk under the potential treatment A = 1, ie, P(Y(1) = 1|A = 1,**W**) equals the one under treatment A = 0, ie, P(Y(1) = 1|A = 0,**W**). In other words: the risk of death for those treated would have been the same as for those untreated if untreated subjects had received, contrary to the fact, the treatment. This is equivalent to assuming that all confounders have been measured. (ii) We also assume that within strata of **W**, every patient had a nonzero probability of receiving either of the 2 treatment conditions, ie, 0 < P(A = 1|**W**) < 1 (positivity). (iii) We assume consistency, which states that we observe the potential outcome corresponding with the observed treatment, ie, for any individual, Y = AY(1) + (1 − A)Y(0). Also, (iv) in defining an individual's counterfactual outcome as only a function of their own treatment, we assume noninterference, meaning that the counterfactual outcome of one subject was not influenced by the treatment of any other. Under the causal assumptions explained above, the ATE may be consistently estimated in the nonparametric model (that does not impose assumptions on either E(Y|A,**W**) or P(A = 1|**W**)). Thus, if we believe these assumptions to hold and the sample size to be sufficient, we may interpret our estimate of the ATE approximately as the marginal risk difference of 1‐year mortality for cancer patients treated with monotherapy versus dual therapy.

### 2.1 Data generation process

**Figure 1:**
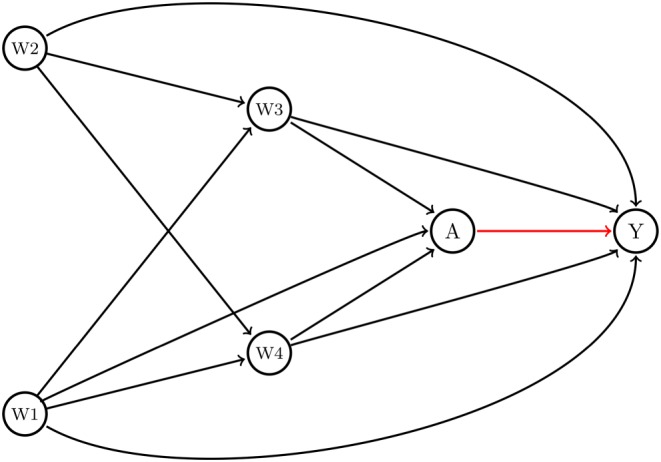
Direct acyclic graph. Legend: Conditional exchangeability of the treatment effect or exposure (A) on cancer mortality (Y) is obtained through conditioning on a set of available covariates (Y(1),Y(0) ⊥ A|**W**). The average treatment effect for the structural framework is estimated as the average risk difference between the expected effect of the treatment conditional on **W** among those treated (E(Y|A = 1; **W**)) and the expected effect of the treatment conditional on **W** among those untreated (E(Y|A = 0; **W**)). Y: mortality binary indicator (1 death, 0 alive), A: binary treatment for cancer with monotherapy versus dual therapy (1 Mono; 0 Dual); **W:** *W*_1_: sex; *W*_2_: age at diagnosis; *W*_3_: cancer stage, TNM classification; *W*_4_: comorbidities [Colour figure can be viewed at http://wileyonlinelibrary.com]

To demonstrate the implementation of TMLE, we generated data, denoted as the set of i.i.d variables O = (**W** = (*W*_1_, *W*_2_, *W*_3_, *W*_4_), A, Y), based on the directed acyclic graph^1^ illustrated in Figure 1.

Briefly, sex (*W*_1_) and age category (*W*_2_) were generated as Bernoulli variables with probabilities 0.5 and 0.65, respectively. Cancer stage (*W*_3_) and comorbidities (*W*_4_) were generated as ordinal variables with 4 and 5 levels, respectively. For both variables, we used a random uniform distribution with minimum 0 and maximum 4 and 5, respectively, and rounded the generated numbers off to the closest integer. The treatment variable (A) and the potential outcomes (Y(1) and Y(0)) were generated as binary indicators using log‐linear models. In the treatment and outcome models, we included an interaction term between age (*W*_2_) and comorbidities (*W*_4_) based on plausible biological mechanisms such as an increased risk of comorbidities among older adults.^26^ Another, more complex data generating process can be found in Appendix S1.

The data‐generating model for the treatment was selected to create the potential for near‐practical positivity violations. In particular, given the possible covariate values and the coefficients, the minimum possible true probability of receiving treatment was 0.002. Finally, for simplicity, we did not include effect modification (ie, interaction between A and **W**) though both TMLE and AIPTW can easily handle this setting as well.

First, we generated a sample of 5 million patients to estimate the true ATE and MOR. Afterwards, we generated a sample of 10 000 patients used to illustrate the implementation of the algorithm and run simulations. In Box 1, we introduce the specifications for the data generation and the true values for the ATE and the MOR. The true ATE implies that the risk of death among cancer patients treated with monotherapy is approximately 19.3% higher than for those treated with dual therapy. The true MOR implies that there is a 2.5 times higher odds of death among cancer patients treated with monotherapy versus dual therapy. Note that for educational purposes, we present the code and results for a single dataset simulated by our data‐generating mechanism. However, at the end of the illustration, we also present the results of 1000 Monte Carlo simulations with a sample size of 1000 patients aiming (i) to estimate the bias of the estimators under near‐positivity violations and mild misspecification of the treatment and outcome models and (ii) to demonstrate TMLE's double‐robustness property by misspecifying the model for the outcome.

Box 1 **Data generation**




# Function to generate data

generateData <- function(n) {  w1 <- **rbinom**(n, size = 1, prob = 0.5)

w2 <- **rbinom**(n, size = 1, prob = 0.65)

w3 &lt;- **round**(**runif**(n, min = 0, max = 4), digits = 0)

w4 &lt;- **round**(**runif**(n, min = 0, max = 5), digits = 0)

A <- **rbinom**(n, size = 1, prob = <b>plogis</b>(-5 + 0.05*w2 + 0.25*w3 + 0.6*w4 + 0.4*w2*w4))

# counterfactual

Y.1 &lt;- **rbinom**(n, size = 1, prob = <b>plogis</b>(-1 + 1 - 0.1*w1 + 0.35*w2 + 0.25*w3 + 0.20*w4 + 0.15*w2*w4))

Y.0 &lt;- **rbinom**(n, size = 1, prob = <b>plogis</b>(-1 + 0 - 0.1*w1 + 0.35*w2 + 0.25*w3 + 0.20*w4 + 0.15*w2*w4))

# observed outcome

Y &lt;- Y.1*A + Y.0*(1 - A)

#return data.frame

data.frame(w1, w2, w3, w4, A, Y, Y.1, Y.0)

}



# True ATE and OR

**set.seed**(7777)

ObsData &lt;- **generateData**(n = 5000000)

True_EY.1 &lt;- **mean**(ObsData$Y.1)

True_EY.0 &lt;- **mean**(ObsData$Y.0)

True_ATE &lt;- True_EY.1 - True_EY.0; True_ATE

True_MOR &lt;- (True_EY.1*(1 - True_EY.0))/((1 - True_EY.1)*True_EY.0); True_MOR



#**True_ATE:  19.3%**

#**True_MOR: 2.5**



# Data for simulation

set.seed(7722)

ObsData &lt;- **generateData**(n = 10000)

In Box 1, **plogis(x)** represents the inverse logit function: $\frac{\exp\left(x\right)}{1+\exp\left(x\right)}$.

## 3 TMLE ALGORITHM: GUIDED IMPLEMENTATION

In this section, we introduce the step‐by‐step implementation of the TMLE algorithm in a set of 10 boxes with documented R code for educational reproducibility.

### 3.1 TMLE implementation

Targeted maximum likelihood estimation can either be used by means of the ***tmle*** function from the R‐package ***tmle*** or by computing the algorithm in 6 steps manually. We first show the latter option for educational purposes; a sound understanding of TMLE will enable the reader to make the right decisions for their analysis.

First step: Prediction of the outcome model bar ${\bar{Q}}^{0}$(A,**W**) = E(Y|A,W)

The superscript 0 in ${\bar{Q}}^{0}\left(A,W\right)$ indicates that it is the initial estimate of E(Y|A,**W**), which is the conditional mean of the outcome (Y) given treatment A and baseline covariates **W**. To obtain ${\bar{Q}}^{0}$(A,**W**), we can use a standard logistic regression model:

$$\text{logit}\left[P\left(Y=1|A,W\right)\right]=\beta_{0}+\beta_{1}A+\beta_{2}^{T}\;W$$

where **logit(x)** represents the function log $\left(\frac{x}{1‐x}\right)$ and **β** is the vector of the estimates for the coefficients of the model parameters. As before, A represents the treatment and **W** is the transpose of the vector of covariates **W** = (*W*_1_, …, *W*_4_) presented in the DAG (Figure 1).

Therefore, we can estimate the initial probabilities as the prediction of this logistic outcome regression model for A = 0 and A = 1, respectively (Box 2):

$$\begin{array}{l} {\bar{Q}}^{0}\left(0,W\right)=\text{expi}t\left({\hat{\beta }}_{0}+{\hat{\beta }}_{1}0+{\hat{\beta }}_{2}^{T}\;W\right)\;\text{and}\\ {\bar{Q}}^{0}\left(1,W\right)=\text{expit}\left({\hat{\beta }}_{0}+{\hat{\beta }}_{1}1+{\hat{\beta }}_{2}^{T}\;W\right), \end{array}$$

where **expit(x)** represents the inverse logit function $\frac{\exp\left(x\right)}{1+\exp\left(x\right)}$.

Note that for educational purposes, we misspecified both the propensity score and outcome models by not accounting for the interaction between age (*W*_2_) and comorbidities (*W*_4_). This allows us to later show the added value of incorporating machine‐learning algorithms for prediction.

Box 2 **Prediction of ${\bar{Q}}^{0}\left(A,W\right)$
**




#First estimation of E(Y|A, **W**), namely 
${\bar{Q}}^{0}\left(A,W\right)$

m &lt;- **glm**(Y ~ A + w1 + w2 + w3 + w4, family = binomial, data = ObsData) #Misspecified model



#Prediction for A, A = 1 and, A = 0

QAW = **predict**(m, type = "response")

Q1W = **predict**(m, newdata = data.frame(A = 1, ObsData[,c("w1","w2","w3","w4")]), type = "response")

Q0W = **predict**(m, newdata = data.frame(A = 0, ObsData[,c("w1","w2","w3","w4")]), type = "response")



#Estimated mortality risk difference (G‐computation)

**mean**(Q1W - Q0W)

#Initial ATE estimate: **20.4%**



#Estimated MOR (G-computation)

**mean**(Q1W)*(1 - **mean**(Q0W)) / ((1 - **mean**(Q1W))*mean(Q0W))

#Initial MOR estimate: **2.7**

As computed at the bottom of Box 2, the G‐computation (untargeted) estimate is the mean of ${\bar{Q}}^{0}\left(1,W\right)\;-\;{\bar{Q}}^{0}\left(0,W\right)$, taken over all subjects. In this case, this is a very good estimate of the ATE and the MOR because the outcome model is nearly correctly specified.

Second step: Prediction of the propensity score $\hat{g}\left(A,W\right)$

In this step, we estimate P(A = 1|**W**), the probability of the treatment with monotherapy versus dual therapy (A) given the set of covariates (**W**), namely, the propensity score. Using a logistic regression model, we set

$$\text{logit}\left[P\left(A=1|W\right)\right]=\alpha_{0}+\alpha_{1}^{T}W.$$

We then estimate the probabilities $\hat{g}\left(1,W\right)$ using

$$\hat{g}\left(1,W\right)=\text{expit}\;\left({\hat{\alpha }}_{0}+{\hat{\alpha }}_{1}^{T}\;W\right)$$

where ${\hat{\alpha }}_{0}$ and ${\hat{\alpha }}_{1}$ are the estimates of the coefficients in the logistic regression.

Box 3 **Prediction of the propensity score $\hat{g}\left(1,W\right)$
**




psm &lt;- **glm**(A ~ w1 + w2 + w3 + w4, family = binomial, data = ObsData) #Misspecified model

gW = **predict**(psm, type = "response") #propensity score values



#Propensity score distribution

<b>summary</b>(gW**)**

Min.1st Qu.Median Mean 3rd Qu.Max.

0.0020.030.0860.1550.2400.718

Note that as in the previous step, here in Box 3, we intentionally did not account for the interaction between age (*W*_2_) and comorbidities (*W*_4_) in the treatment model. Also, the summary of the estimated propensity score (g**W**) indicates near violations of the practical positivity assumption given the very small numbers for the lower tail of the distribution, an interesting setting under which to compare TMLE with AIPTW.

The next 2 steps aim to improve the prediction model ${\bar{Q}}^{0}\left(A,W\right)$ from step 1 by incorporating information from the propensity score. This can be viewed as updating ${\bar{Q}}^{0}\left(A,W\right)$ along a path to incorporate additional information from the propensity score function with the goal of reducing the residual confounding in the first estimate of ${\bar{Q}}^{0}\left(A,W\right)$.

Third step: The clever covariate and estimation of ε

This step aims to compute the clever covariates H(1, **W**) and H(0, **W**), and a vector fluctuation parameter called ε. The clever covariates in Equation (1) are calculated for each individual in the data, based on their previously estimated probabilities of exposure status, P(A = 1|**W**) and P(A = 0|**W**). Note that the functional forms of the clever covariates are very similar to inverse probability of treatment weights:

(1) $$H\left(1,W\right)=\frac{A}{\hat{g}\left(1,W\right)};\;H\left(0,W\right)=\frac{1‐A}{\hat{g}\left(0,W\right)}.$$

The fluctuation parameter $\hat{\varepsilon }=\left({\hat{\varepsilon }}_{0},{\hat{\varepsilon }}_{1}\right)$ is estimated through a maximum likelihood procedure; we set a model with the observed outcome (Y) as dependent variable and the logit of the initial prediction of ${\bar{Q}}^{0}\left(A,W\right)$ as an offset (fixed quantity) in an intercept‐free logistic regression with the clever covariates, H(1,**W**) and H(0,**W**) as independent variables (Box 4):

(2) $$E\left(Y=1|\;A,W\right)\left(\varepsilon \right)=\frac{1}{1+\exp\left(-\log\frac{{\bar{Q}}^{0}\left(A,W\right)}{\left(1-{\bar{Q}}^{0}\left(A,W\right)\right)}\;-\;\varepsilon_{0}H\left(0,W\right)\;-\;\varepsilon_{1}H\left(1,W\right)\right)}.$$

When there is little variability in $Y-{\bar{Q}}^{0}\left(A,W\right)$ (ie, the outcome minus the offset), the fluctuation parameter will be estimated as close to 0. In the absence of residual confounding, the propensity score does not provide additional information to improve the initial estimate of ${\bar{Q}}^{0}\left(A,W\right)$ because ${\bar{Q}}^{0}\left(A,W\right)$ was correctly specified. Given a misspecified ${\bar{Q}}^{0}\left(A,W\right),$ the fluctuation parameter may also be estimated as approximately 0 when $\hat{g}\left(1,W\right)$ has no supplementary information relevant to Y. However, TMLE is consistent as long as either the model or the treatment model is estimated consistently. The clever covariates are called “clever” because their form guarantees that the estimating equation corresponding to the efficient IC is solved, which in turn yields desirable (asymptotic) inferential properties, including double‐robustness and local efficiency. A complete explanation of how the clever covariates are chosen is given in van der Laan and Rose,^10^ Chapter 5.

In Box 4, we show how to compute the clever covariates and how to estimate ε. Note that **qlogis(x)** represents the logit function in R.

Box 4 **Computation of the clever covariates, H(1, W) and H(0, W), and estimation of $\hat{\varepsilon }$
**




#Clever covariate and fluctuating/substitution parameters

H1W = (ObsData$A / gW)

H0W = (1 - ObsData$A) / (1 - gW)

epsilon &lt;- coef(**glm**(ObsData$Y ~ -1 + H0W + H1W + offset(**qlogis**(QAW)), family = binomial)); epsilon



**#epsilon**: **0.003, 0.003**

Alternatively, one could add the clever covariates not as covariates, but as weights in this same update step. This is another valid targeted maximum likelihood estimator, based on a refined loss function (and parametric sub model), and can improve stability.^10^

Fourth step: Update of ${\bar{Q}}^{0}\left(A,W\right)\;\text{to}\;{\bar{Q}}^{1}\left(A,W\right)$

The updated estimate of E(Y|A,**W**) is denoted ${\bar{Q}}^{1}\left(A,W\right)$. This update is performed by plugging in $\hat{\varepsilon }=\left({\hat{\varepsilon }}_{0},{\hat{\varepsilon }}_{1}\right)$ into the following equations (Box 5):

(3) $${\bar{Q}}^{1}\left(0,W\right)=\text{expit}\left[\text{logit}\left({\bar{Q}}^{0}\left(0,W\right)\right)+{\hat{\varepsilon }}_{0}/\hat{g}\left(0,W\right)\right]\;\text{and}\;{\bar{Q}}^{1}\left(1,W\right)=\text{expit}\left[\text{logit}\left({\bar{Q}}^{0}\left(1,W\right)\right)+{\hat{\varepsilon }}_{1}/\hat{g}\left(1,W\right)\right].$$

In this way, the update is done separately under the treatment and under the control by evaluating both of these expressions for all subjects.

Fifth step: Targeted estimate of the ATE and the MOR

Finally, the ATE and MOR are estimated as follows:

(4) $${\hat{\mathrm{ATE}}}_{\text{TMLE}}=\frac{1}{n}\overset{n}{\underset{i=1}{\Sigma }}{\bar{Q}}^{1}\left(1,W_{i}\right)\;-\;{\bar{Q}}^{1}\left(0,W_{i}\right)\;\text{and}$$

(5) $${\hat{\mathrm{MOR}}}_{\text{TMLE}}=\frac{\left\{\left[\frac{1}{n}\Sigma_{i=1}^{n}{\bar{Q}}^{1}\left(1,W_{i}\right)\;\right]x\left[1\;-\;\frac{\;1}{\;n}\Sigma_{i=1}^{n}{\bar{Q}}^{1}\left(0,W_{i}\right)\;\right]\right\}}{\left\{\left[1\;-\;\frac{1}{n}\Sigma_{i=1}^{n}{\bar{Q}}^{1}\left(1,W_{i}\right)\;\right]x\left[\frac{1}{n}\Sigma_{i=1}^{n}{\bar{Q}}^{1}\left(0,W_{i}\right)\right]\right\}},$$

where **W**_i_ denotes the ith individual's vector of covariates.

Box 5 **Update from ${\bar{Q}}^{0}\;\text{to}\;{\bar{Q}}^{1}$ and ATE and MOR estimates**




Q0W_1 &lt;- **plogis**(**qlogis**(Q0W) + epsilon[1] / (1 - gW))

Q1W_1 &lt;- **plogis**(**qlogis**(Q1W) + epsilon[2] / gW)



ATEtmle1 &lt;- **mean**(Q1W_1 - Q0W_1); ATEtmle1

EY1tmle1 &lt;- **mean**(Q1W_1)

EY0tmle1 &lt;- **mean**(Q0W_1)

MORtmle1 &lt;- (EY1tmle1 * (1 - EY0tmle1)) / ((1 - EY1tmle1) * EY0tmle1); MORtmle1



**#ATEtmle1: 22.1%**

**#Marginal Odds Ratio (MORtmle1): 3.0**

Table 1 shows the first 5 rows of the final dataset where the predicted value of ${\bar{Q}}^{1}\left(1,W\right)-{\bar{Q}}^{1}\left(0,W\right)$ for the first patient (id = 1) can be estimated by using Equation (3) as the inverse logit transformation of the initial estimates ${\bar{Q}}^{0}\left(1,W\right)$ and ${\bar{Q}}^{0}\left(0,W\right)$ plus the estimated fluctuation parameter epsilon $\left(\hat{\varepsilon }\right)$ times the clever covariates as follows:

$${\bar{Q}}^{1}\left(1,W_{i}\right)-{\bar{Q}}^{1}\left(0,W_{i}\right)=\text{plogis}\left(\text{qlogis}\left({\bar{Q}}^{0}\left(1,W_{i}\right)\right)+{\hat{\varepsilon }}_{1}/\hat{g}\left(1,W_{i}\right)\right)-\text{plogis}\left(\text{qlogis}\left({\bar{Q}}^{0}\left(0,W_{i}\right)+{\hat{\varepsilon }}_{0}/\hat{g}\left(0,W_{i}\right)\right)\right)$$

**Table 1:** Final dataset for the update of ${\bar{Q}}^{0}\left(A,W\right)$ to ${\bar{Q}}^{1}\left(A,W\right)$

id	Q1W_0_	Q0W_0_	g**W**	Epsilon 1	Epsilon 2	${\bar{Q}}^{1}\left(1,W_{i}\right)-{\bar{Q}}^{1}\left(0,W_{i}\right)$
1	0.8551	0.6702	0.1967	0.003	0.0027	0.1858
2	0.639	0.3787	0.0184	0.003	0.0027	0.2927
3	0.7494	0.5073	0.0509	0.003	0.0027	0.2511
4	0.6604	0.4011	0.0095	0.003	0.0027	0.3187
5	0.9152	0.7879	0.5908	0.003	0.0027	0.1264
…	…	…	…	…	…	…

**plogis**(**qlogis**(Q1W_0) + epsilon[2] / gW) − **plogis**(**qlogis**(Q0W_0) + epsilon[1] / (1‐gW)) ***=*** 0.1858

Sixth step: Statistical inference and 95% CI

Targeted maximum likelihood estimation constructs estimators based on the efficient IC,^19^, ^20^ which can be used to obtain standard errors. Based on semiparametric and empirical processes theory, the IC of a consistent and asymptotically linear estimator is derived in the gradient of the pathwise derivative of the target parameter such that^27^, ^28^

(6) $$\hat{\mathrm{ATE}}‐\mathrm{ATE}=\frac{1}{n}\overset{n}{\underset{i=1}{\Sigma }}{\mathrm{IC}}_{i}‐O_{p}\left(\frac{1}{\sqrt{n}}\right).$$

By the weak law of the large numbers, the O_p_ term in equation (6) converges to 0 at a rate of $\frac{1}{\sqrt{n}}$ as the sample size (n) goes to infinity.^10^ The IC is a function of the data and the data‐generating components that one can derive for a given model and target parameter that has mean 0 and finite variance. The central limit theorem applies so that in large samples, the variance of the estimator is thus the variance of the IC divided by n. While many influence functions and corresponding estimators exist for a given target parameter, there always exists an “efficient” IC that achieves the lower bound on asymptotic variance for the given set of modelling assumptions. Targeted maximum likelihood estimation and AIPTW are both constructed by using the efficient IC, making these estimators asymptotically efficient for the statistical model when all necessary models are correctly specified. For the ATE, the efficient IC is

$${\mathrm{EIC}}_{\mathrm{ATE}}=\left(\frac{A}{P\left(A=1|W\right)}\;-\;\frac{1-A}{P\left(A=0|W\right)}\;\right)\left[Y-E\left(Y|A,W\right)\right]+E\left(Y|A=1,W\right)-E\left(Y|A=0,W\right)-\mathrm{ATE},$$

which can be evaluated as

(7) $${\hat{\mathrm{EIC}}}_{\mathrm{ATE}}=\left(\frac{A}{\hat{g}\left(1,W\right)}\;-\;\frac{1-A}{\hat{g}\left(0,W\right)}\;\right)\left[Y-{\bar{Q}}^{1}\left(A,W\right)\right]+{\bar{Q}}^{1}\left(1,W\right)-{\bar{Q}}^{1}\left(0,W\right)-{\hat{\mathrm{ATE}}}_{\text{TMLE}}$$

for every subject. Using the resulting vector, the estimation of the standard error for ${\hat{\mathrm{ATE}}}_{\text{TMLE}}$ is done as follows:

(8) $${\hat{\sigma }}_{\mathrm{ATE},\text{TMLE}}=\sqrt{\frac{\hat{\mathrm{Var}}\left({\hat{\mathrm{EIC}}}_{\mathrm{ATE}}\right)}{n}},$$

where $\hat{\mathrm{Var}}\left({\hat{\mathrm{EIC}}}_{\mathrm{ATE}}\right)$ represents the “sample variance” of the estimated IC.

Based on the functional Delta method,^29^ the efficient IC for the MOR is

(9) $${\mathrm{EIC}}_{\mathrm{MOR}}=\frac{1-E\left[Y\left(0\right)\right]}{{\left(1-E\left[Y\left(1\right)\right]\right)}^{2}x\;E\left[Y\left(0\right)\right]}\;\times \;D1\;-\;\frac{E\left[Y\left(1\right)\right]}{\left(1-E\left[Y\left(1\right)\right]\right)x\;{\left(E\left[Y\left(0\right)\right]\right)}^{2}}\;\times \;D0,$$

where D1 and D0 are the efficient IC for E[Y(1)] and E[Y(0)], respectively:

(10) $$D1=\left(\frac{A}{P\left(A=1|W\right)}\;\right)\left[Y-E\left(Y|A=1,W\right)\right]+E\left(Y|A=1,W\right)-E\left[Y\left(1\right)\right],$$

(11) $$D0=\left(\frac{1-A}{P\left(A=0|W\right)}\;\right)\left[Y-E\left(Y|A=0,W\right)\right]+E\left(Y|A=0,W\right)-E\left[Y\left(0\right)\right].$$

In Box 6, it is shown how to evaluate the efficient IC for the ATE and the MOR based on Equations (5) to (11).

Box 6 **Estimation of the SE and 95% CI for the ${\hat{\mathrm{ATE}}}_{\text{TMLE}}$ and ${\hat{\mathrm{MOR}}}_{\text{TMLE}}$
**

**#ATE efficient influence curve (EIC)**

D1 &lt;- ObsData$A/gW*(ObsData$Y - Q1W_1) + Q1W_1 - EY1tmle1

D0 &lt;- (1 - ObsData$A)/(1 - gW)*(ObsData$Y - Q0W_1) + Q0W_1 - EY0tmle1

EIC &lt;- D1 - D0

#ATE variance

n &lt;- **nrow**(ObsData)

varHat.IC &lt;- **var**(EIC)/n

#ATE 95%CI

ATEtmle1_CI &lt;- c(ATEtmle1 - 1.96*sqrt(varHat.IC), ATEtmle1 + 1.96*sqrt(varHat.IC)); ATEtmle1; ATEtmle1_CI



#**ATEtmle1_CI(95%CI): 22.1% (15.1, 29.0)**



**#MOR EIC**

EIC &lt;- (1 - EY0tmle1) / EY0tmle1 / (1 - EY1tmle1)^2 * D1 - EY1tmle1 / (1 - EY1tmle1) / EY0tmle1^2 * D0

varHat.IC &lt;- **var**(EIC)/n

#MOR 95%CI

MORtmle1_CI &lt;- c(MORtmle1 - 1.96*sqrt(varHat.IC), MORtmle1 + 1.96*sqrt(varHat.IC)); MORtmle1; MORtmle1_CI



**#MORtmle1_CI(95%CI): 3.0 (1.6, 4.3)**

### 3.2 Comparative performance of TMLE: TMLE vs naïve logistic regression approach and AIPTW

In Box 7, we show the estimation and results of the naïve logistic regression approach, which assumes a correctly specified parametric outcome model (violated in the example below) and a constant effect of the treatment (A) on 1‐year mortality (not violated in the example below). In addition, because of the noncollapsibility of the odds ratio, even a correctly specified logistic regression model generally does not produce estimates of the MOR.

Box 7 **Estimation of the conditional odds ratio using the naïve logistic regression approach**




Naive &lt;- **glm**(data = **ObsData**, Y ~ A + w1 + w2 + w3 + w4, family = binomial)

summary(Naive)

exp(Naive$coef[2])

exp(confint(Naive))

**#Naïve OR (95%CI): 2.9 (2.5, 3.4)**

AIPTW, which directly uses the efficient IC, can also be used to estimate the ATE and the MOR. The ATE and MOR using AIPTW are computed as follows:

$${\hat{\mathrm{EY}1}}_{\text{AIPTW}}=\frac{1}{n}\overset{n}{\underset{i=1}{\Sigma }}\left\{\frac{I\left(A_{i}=1\right)}{\hat{g}\left(1,W_{i}\right)}\left(Y_{i}\;-\;{\bar{Q}}^{0}\left(1,W_{i}\right)\right)+{\bar{Q}}^{0}\left(1,W_{i}\right)\right\}.$$

$${\hat{\mathrm{EY}0}}_{\text{AIPTW}}=\frac{1}{n}\overset{n}{\underset{i=1}{\Sigma }}\left\{\frac{I\left(A_{i}=0\right)}{\hat{g}\left(0,W_{i}\right)}\left(Y_{i}\;-\;{\bar{Q}}^{0}\left(0,W_{i}\right)\right)+{\bar{Q}}^{0}\left(0,W_{i}\right)\right\}.$$

(12) $${\hat{\mathrm{ATE}}}_{\text{AIPTW}}={\hat{\mathrm{EY}1}}_{\text{AIPTW}}\;-\;{\hat{\mathrm{EY}0}}_{\text{AIPTW}}.$$

(13) $${\hat{\mathrm{MOR}}}_{\text{AIPTW}}=\frac{{\hat{\mathrm{EY}1}}_{\text{AIPTW}}\left(1\;-\;{\hat{\mathrm{EY}0}}_{\text{AIPTW}}\right)}{\left(1-{\hat{\mathrm{EY}1}}_{\text{AIPTW}}\right){\hat{\mathrm{EY}0}}_{\text{AIPTW}}},$$

where **W**_i_ denotes the ith individual's vector of covariates.

In Box 8, we provide code for the estimation of the ATE and the MOR (with variances and CIs) using AIPTW.

Box 8 **Estimation of the ATE and MOR using AIPTW**




EY1aiptw &lt;- **mean**((ObsData$A) * (ObsData$Y - Q1W) / gW + Q1W)

EY0aiptw &lt;- **mean**((1 - ObsData$A) * (ObsData$Y - Q0W) / (1 - gW) + Q0W)



AIPTW_ATE &lt;- EY1aiptw - EY0aiptw; AIPTW_ATE

AIPTW_MOR &lt;- (EY1aiptw * (1 - EY0aiptw)) / ((1 - EY1aiptw) * EY0aiptw); AIPTW_MOR



**#Calculation of the efficient IC**

D1 &lt;- (ObsData$A) * (ObsData$Y - Q1W) / gW + Q1W - EY1aiptw

D0 &lt;- (1 - ObsData$A) * (ObsData$Y - Q0W) / (1 - gW) + Q0W - EY0aiptw

varHat_AIPTW &lt;- **var**(D1 - D0) / n



ATEaiptw_CI &lt;- c(AIPTW_ATE - 1.96*sqrt(varHat_AIPTW), AIPTW_ATE + 1.96*sqrt(varHat_AIPTW)); AIPTW_ATE; ATEaiptw_CI

**#ATEaiptw_CI(95%CI): 24.0% (16.4, 31.6)**



ICmor_aiptw &lt;- (1 - EY0aiptw) / EY0aiptw / (1 - EY1aiptw)^2 * D1 - EY1aiptw / (1 - EY1aiptw) / EY0aiptw^2 * D0

varHat_AIPTW2 &lt;- **var**(ICmor_aiptw) / n



MORaiptw_CI &lt;- c(AIPTW_MOR - 1.96*sqrt(varHat_AIPTW2), AIPTW_MOR + 1.96*sqrt(varHat_AIPTW2)); AIPTW_MOR; MORaiptw_CI



**#MORaiptw_CI(95%CI): 3.4 (1.6, 5.2)**

### 3.3 TMLE improved performance calling the Super‐Learner

Note that in the one‐sample simulation illustrated so far, we have intentionally misspecified both models (treatment and outcome) to allow for evaluation of the benefit of calling the *SuperLearner* (SL) R package (Boxes 9 and 10). The SL, which is a type of *ensemble learner*, adaptively combines different machine‐learning algorithms to (separately) estimate ${\bar{Q}}^{0}\left(A,W\right)$ and $\hat{g}\left(A,W\right)$. The aim of including this approach in the TMLE algorithm is to avoid bias arising from model misspecification.^10^ SL thus replaces the simple logistic models in steps 1 and 2 above. One generally selects a set of prediction algorithms for use in the SL, which may include parametric regression models, nonlinear regression models, shrinkage estimators, and regression trees. Instead of choosing the algorithm with the smallest expected prediction error (estimated by cross validation), the SL selects a weighted combination of different algorithms. Specifically, it selects the weighted combination of predictions that minimises the cross‐validated mean square error (Supplementary Figure S1).^30^ It can be shown that this weighted combination will perform asymptotically at least as well as the best algorithm (in the cross‐validated error), but typically even better.^10^, ^19^, ^23^, ^30^ Box 9 describes the implementation of TMLE using the R‐package “***tmle***” while calling the SL. The basic implementation of TMLE in the R‐package ***tmle*** uses by default 3 algorithms: “SL.glm” (logistic regression using A and **W** as covariates), “SL.step” (stepwise model selection of **W** in a generalized linear model using Akaike's information criterion to determine subsets of the full model), and “SL.glm.interaction” (a generalised linear model that includes all 2‐way interactions of the terms included in the full model).^23^ To list all implemented algorithms, one can simply type “listWrappers()” in R. In Box 10, we illustrate the reduction of bias by calling, in addition to default algorithms implemented in ***tmle***, more advanced machine‐learning algorithms such as generalised additive models, random forests, and recursive partitioning. For a more advanced theoretical background regarding machine‐learning algorithms, see Hastie et al.^31^

Box 9 **TMLE using default implementation with the default SL library**




library(**tmle**)

library(**SuperLearner**)

TMLE2 &lt;- **tmle**(Y = ObsData$Y, A = ObsData$A, W = ObsData[,c("w1", "w2", "w3", "w4")], family = "binomial")



ATEtmle2 &lt;- TMLE2$estimates$ATE$psi;ATEtmle2

TMLE2$estimates$ATE$CI

MORtmle2 &lt;- TMLE2$estimates$OR$psi;MORtmle2

TMLE2$estimates$OR$CI

#**ATEtmle2 (95%CI): 20.8% (17.5, 24.1)**

**#MORtmle2 (95%CI): 2.8 (2.3, 3.4)**

Box 10 **TMLE with a user‐selected SL library**




library(**tmle**)

library(**SuperLearner**)



SL.library &lt;- c("SL.glm","SL.step","SL.step.interaction", "SL.glm.interaction","SL.gam",

"SL.randomForest", "SL.rpart")



TMLE3 &lt;- **tmle**(Y = ObsData$Y,A = ObsData$A,W = ObsData [,c("w1", "w2", "w3", "w4")],

family = "binomial", Q.SL.library = SL.library,g.SL.library = SL.library)



ATEtmle3 &lt;- TMLE3$estimates$ATE$psi;ATEtmle3

TMLE3$estimates$ATE$CI

MORtmle3 &lt;- TMLE3$estimates$OR$psi;MORtmle3

TMLE3$estimates$OR$CI



#**ATEtmle3 (95%CI): 20.7% (17.5, 24.0)**

#**MORtmle3 (95%CI): 2.7 (2.2, 3.3)**

**Figure 2:**
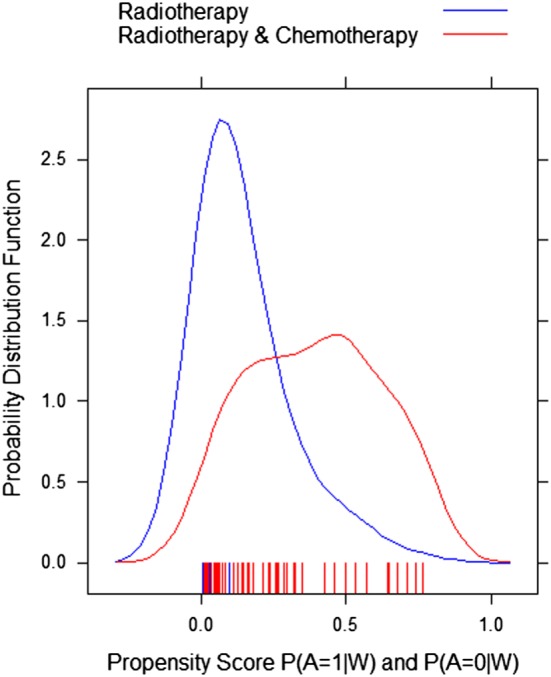
Probability density function of the propensity score by treatment status for one randomly selected sample from 1000 Monte Carlo simulations [Colour figure can be viewed at http://wileyonlinelibrary.com]

Table 2 summarises the results of 1000 Monte Carlo simulations each with a sample size of 1000 patients based on the data generation process introduced in Box 1. We present the ATE and MOR estimates for 3 different estimators (naïve regression, AIPTW, and TMLE) and 3 different versions of TMLE (TMLE‐1‐2‐3, Table 2) corresponding to the usage of either logistic regressions or SL, implemented either with the default or user‐supplied library given in Box 10, for the estimation of the outcome expectation and the propensity score. Furthermore, our data generation often produced near‐practical positivity violations (ie, certain subgroups in the samples rarely or never received treatment) as described above. We checked the violation visually (Figure 2) and numerically, based on the empirical summary of the propensity score distribution (mean = 0.189, min = 0.002 and max = 0.765). In the logistic regressions, the models for the treatment and the outcome were misspecified by omitting the interactions between age and comorbidities. Finally, to demonstrate the TMLE double‐robustness property, we ran a second set of simulations when correctly specifying the propensity score using the true logistic regression model (with the outcome model either incorrectly specified with a main term logistic regression or adaptively estimated with SL). Complete details of the models' functional forms for the data generation and the full settings for the simulation are provided in the Appendix S1 and at the following GitHub repository: https://github.com/migariane/SIM-TMLE-tutorial, making our results fully reproducible.

**Table 2:** ATE and COR Monte Carlo simulations for mild misspecified models and near‐positivity violation, n = 1000

Misspecified treatment and outcome models		Naïve	AIPTW	TMLE‐1	TMLE‐2	TMLE‐3
True ATE	0.193					
Estimate ATE			0.208	0.199	0.193	0.193
Absolute bias ATE			0.015	0.006	0.000	0.000
Relative bias ATE (%)			7.2%	3.0%	0.0%	0.0%
True MOR	2.5					
Estimate MOR		3.1	3.0	3.0	2.9	2.8

Treatment model correctly specified						
True ATE	0.224					
Estimate ATE			0.226	0.226	0.225	0.224
Absolute bias ATE			0.002	0.002	0.001	0.000
Relative bias ATE (%)			0.8%	0.8%	0.4%	0.0%
True MOR	2.6					
Estimate MOR		2.9	2.8	2.8	2.8	2.7

$\hat{\mathrm{ATE}}$ **:** Estimated average treatment effect from the 1000 simulation repetitions.

$\hat{\mathrm{MOR}}$ **:** Estimated marginal odds ratio from the 1000 simulation repetitions.

**Naïve:** Logistic regression.

**AIPTW:** Augmented inverse‐probability treatment weights estimation under dual misspecification (model for the treatment and the outcome).

**TMLE‐1:** Dual misspecification. Algorithm computed by hand and naïve prediction (using from logistic regression models) without Super‐Learner (SL).

**TMLE‐2:** Dual misspecification. Algorithm estimated using R‐package ***tmle*** and default SL library (SL.glm, SL.step, and SL.glm.interaction).

**TMLE‐3:** Dual misspecification. Algorithm computed using R‐package ***tmle,*** user‐supplied SL library (SL.gam, SL.randomForest, and SL.rpart).

Treatment model correctly specified refers to the usage of the correct logistic regression model for the propensity score. For TMLE‐2 and TMLE‐3, SL is used to estimate the outcome model as in the first scenario.

In this example, the true ATE is 19.3% and the MOR of monotherapy versus dual therapy was 2.5. The results of the different estimators are presented in Table 2. The naïve approach overestimated the MOR by 24%, whereas the AIPTW and TMLE‐1 overestimated it by 20%, likely because of model misspecification. However, TMLE‐3, which used a more diverse SL library, reduced the bias for the MOR to 12%. Regarding the simulation results for the risk differences, the AIPTW estimator overestimated the ATE by 7%, whereas TMLE‐1 overestimated it by just 3%. However, TMLE‐2 and TMLE‐3 reduced the bias for the ATE to 0%. Therefore, the introduction of machine‐learning algorithms and ensemble learning as discussed above reduced the bias of TMLE‐2 and TMLE‐3. Finally, under misspecification of the outcome model, but correct specification of the propensity score model, the TMLE‐3 was unbiased for the ATE and the MOR.

## 4 DISCUSSION

The quantity, quality, and variety of available data for observational epidemiology continue to grow extensively, making the estimation of causal effects more accessible and popular among applied statisticians and epidemiologists. Targeted maximum likelihood estimation implemented with ensemble and machine‐learning algorithms has advantages over other methods, but surprisingly there is limited guidance for the application of the technique for the estimation of the ATE and MOR when dealing with binary outcomes.^32^ By using a reproducible example, we have demonstrated that the implementation of the TMLE algorithm is not a black box. Using a guided step‐by‐step approach, we have provided the basic elements needed to understand how TMLE works. Furthermore, we have demonstrated the positive benefit, in bias reduction and performance, of adding more advanced machine‐learning algorithms to the fitting process of TMLE. Our educational example adds to other already available tutorials,^32^, ^33^, ^34^, ^35^, ^36^ but with a different motivation based on population‐based cancer epidemiology, a clear orientation towards applied statisticians, and fully explained and available code to replicate step‐by‐step the implementation of TMLE in both Stata and R statistical software. However, our simulation scenarios were relatively simple. We simulated categorical and binary variables and did not produce interaction between the treatment and the vector of confounders, which might not be a realistic setting. Allowing for effect modification (interaction between A and **W**) is an interesting case, because this is a specific setting where the naïve regression adjustment does not work. Despite that, the main interest of the illustration was to introduce the step‐by‐step implementation of the TMLE algorithm and demonstrate the added value of adding machine and ensemble learning algorithms for prediction. Readers will find an additional example with treatment effect modification in Appendix S1 where we used the R‐package ***simcausal*.**

The implementation of TMLE for the estimation of the ATE with a continuous outcome follows identical steps to those listed above. However, note that for this case, (i) we first transform the outcome via Y − a/(b − a), where a and b are plausible values for lower and upper limits of Y; (ii) proceed with the steps from the tutorial; and (iii) rescale the estimate of the ATE and the variance at the end. Readers can find additional depth including the mathematical derivation of TMLE in Chapter 7 of the Targeted Learning book.^10^

Adding more adaptive SuperLearner algorithms requires a significant amount of computer memory and computational time. Hence, with large sample sizes, such as those in population‐based cancer research, TMLE will benefit from additional computing resources such as a cluster environment. For instance, for a computer with 1 core and 16 GB of memory, the R‐package ***tmle*** took 5.4 minutes to estimate the ATE for 10 000 patients using more advanced machine‐learning algorithms including generalised additive models, random forests, and boosting. Furthermore, some machine‐learning algorithms will not work properly depending on the nature of the observed data (eg, for small sample sizes or nonbinary outcomes). Moreover, some theoretical background knowledge of machine learning is required to decide for the most adaptive SL algorithms.

Using TMLE in practical settings is similar to the strategy used for analysts when applying other double‐robust methods such as AIPTW (eg, summarising the inverse probability of treatment weights, checking the distribution of the weights by levels of the treatment, and considering if appropriate the truncation of the weights). Also, we suggest considering the extent to which the data allow for very complicated machine‐learning algorithms, a question closely related with the dimensionality of the data (ie, for small datasets and continuous outcomes, some machine learning might not perform well). Finally, we suggest that readers aiming to develop TMLE approaches follow the causal roadmap introduced above and described by Van der Laan and coauthors.^10^ Similarly, readers can find additional depth including the mathematical derivation of TMLE in Chapter 5 of the Targeted Learning book.^10^

It is important to mention that TMLE is a general template for the construction of efficient substitution estimators for a given estimation problem defined by a statistical model and a targeted parameter. Targeted maximum likelihood estimation is a very active research area, and many advances have been and are currently being made. Clearly, these advances have not been covered here in our introductory tutorial, but a reader might be interested in such topics as the TMLE for rare outcomes,^37^ the Collaborative TMLE for the cross‐validated conditional risk of a candidate estimator,^38^, ^39^, ^40^ and the TMLE for longitudinal settings where time‐dependent confounding is an issue.^34^, ^41^, ^42^, ^43^

In summary, we have provided an accessible presentation with documented code for implementing TMLE to estimate the ATE and MOR for a binary outcome in observational studies. Given TMLE's appealing statistical properties, we consider it a suitable method to be added to the analytical toolbox for estimation of causal effects in large population‐based observational studies.

## AUTHORS' CONTRIBUTIONS

MALF developed the concept and design of the study. MALF carried out the simulations and analysed the data and wrote the manuscript. All authors interpreted the data, drafted and revised the manuscript, code, and results critically. All authors read and approved the final version of the manuscript. MALF is the guarantor of the paper.

## Supporting information

Figure S1. Illustration of Super‐learner algorithm and ensemble learning technique

Data S2. Appendix

Appendix S1
