# Supplementary material for: Targeted maximum likelihood estimation for a binary treatment: A tutorial
Source: Stat Med. 2018 Apr 23;37(16):2530–46. doi: 10.1002/sim.7628 (PMC6032875; doi:10.1002/sim.7628)
Supplement: Supplementary file 1 — Figure S1. Illustration of Super‐learner algorithm and ensemble learning technique [file SIM-37-2530-s001.docx]

**Supplementary Figure 1.** Illustration of Super-learner algorithm and ensemble learning technique.
