# Supplementary material for: Targeted maximum likelihood estimation for a binary treatment: A tutorial
Source: Stat Med. 2018 Apr 23;37(16):2530–46. doi: 10.1002/sim.7628 (PMC6032875; doi:10.1002/sim.7628)
Supplement: Supplementary file 2 — Data S2. Appendix [file SIM-37-2530-s002.pdf]

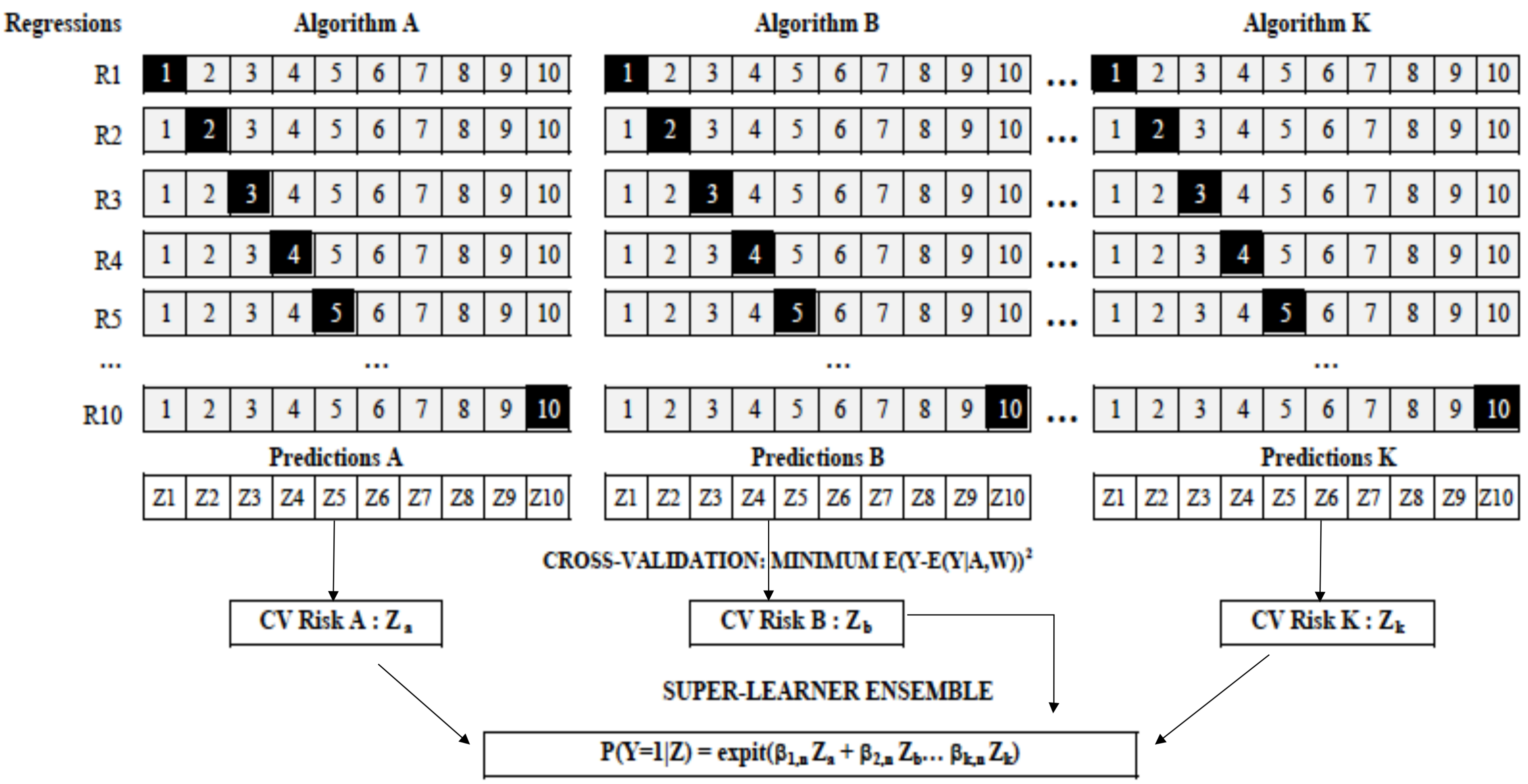

Regressions
Algorithm A
Algorithm B
Algorithm K
R1
R2
R3
R4
R5
...
R10
1 2 3 4 5 6 7 8 9 10
Predictions A
Predictions B
Predictions K
Z1 Z2 Z3 Z4 Z5 Z6 Z7 Z8 Z9 Z10
CROSS-VALIDATION: MINIMUM E(Y-E(Y|A,W))²
CV Risk A : Za
CV Risk B : Zb
CV Risk K : Zk
SUPER-LEARNER ENSEMBLE
P(Y=1|Z) = expit(β1,n Za + β2,n Zb… βk,n Zk)
