# Supplementary material for: Targeted maximum likelihood estimation for a binary treatment: A tutorial
Source: Stat Med. 2018 Apr 23;37(16):2530–46. doi: 10.1002/sim.7628 (PMC6032875; doi:10.1002/sim.7628)
Supplement: Supplementary file 3 — Appendix S1 [file SIM-37-2530-s003.pdf]

**APPENDIX**

# Targeted Maximum Likelihood Estimation for a Binary Treatment: A Tutorial

**AUTHORS**

Miguel Angel Luque-Fernandez*[1,2,3], Michael Schomaker[4], Bernard Rachet[1], Mireille E. Schnitzer[5]

**AUTHOR'S AFFILIATIONS**

1. Faculty of Epidemiology and Population Health. Department of Non-Communicable Disease Epidemiology. Cancer Survival Group. London School of Hygiene and Tropical Medicine, London, U.K.
2. Harvard T.H. Chan School of Public Health. Department of Epidemiology. Boston, U.S.
3. Andalusian School of Public Health. Non-Communicable and Cancer Epidemiology Group (ibs.Granada), Granada, Spain
4. The University of Cape Town, School of Public Health and Family Medicine, Center for Infectious Disease Epidemiology and Research. Cape Town, South Africa
5. Université de Montréal, Faculté de pharmacie, Montréal, Canada

***Corresponding author:**

Miguel Angel Luque-Fernandez, PhD
Keppel Street, London WC1E 7HT
Phone: +4402079588162
miguel-angel.luque@lshtm.ac.uk

**APPENDIX**

**Content**

1. TMLE with R: code for illustration
2. Stata code for manual implementation of TMLE
3. Link to a GitHub repository for the implementation of TMLE in Stata
4. R code for simulations (Manuscript Table 2)
5. Super Learner, cross-validation and ensemble learning

## 1. TMLE with R: code for the illustration

```
##########################################################################################
# Miguel Angel Luque Fernandez, Michael Schomaker, Bernard Rachet, Mireille Schnitzer
# Targeted Maximum Likelihood Estimation for a Binary treatment: A tutorial
# R-syntax included in the boxes of the manuscript
##########################################################################################

# Function to generate data (DGP)
generateData<- function(n){
  w1 <- rbinom(n, size=1, prob=0.5)
  w2 <- rbinom(n, size=1, prob=0.65)
  w3 <- round(runif(n, min=0, max=4), digits=0)
  w4 <- round(runif(n, min=0, max=5), digits=0)
  A <- rbinom(n, size=1, prob= plogis(-5 +  0.05*w2 + 0.25*w3 + 0.6*w4 + 0.4*w2*w4))
  # counterfactual
  Y.1 <- rbinom(n, size=1, prob= plogis(-1 + 1 -0.1*w1 + 0.35*w2 + 0.25*w3 + 0.20*w4 + 0.15*w2*w4))
  Y.0 <- rbinom(n, size=1, prob= plogis(-1 + 0 -0.1*w1 + 0.35*w2 + 0.25*w3 + 0.20*w4 + 0.15*w2*w4))
  # Observed outcome
  Y <- Y.1*A + Y.0*(1 - A)
  # return data.frame
  data.frame(w1, w2, w3, w4, A, Y, Y.1, Y.0)
}
# True ATE in the population
set.seed(7777)
ObsDataTrueATE <- generateData(n = 5000000)
True_EY.1 <- mean(ObsDataTrueATE$Y.1)
True_EY.0 <- mean(ObsDataTrueATE$Y.0)
True_ATE  <- True_EY.1-True_EY.0 ;True_ATE
True_MOR  <- (True_EY.1*(1-True_EY.0))/((1-True_EY.1)*True_EY.0);True_MOR

cat("\n True_ATE:", abs(True_ATE))

# Data for analysis
set.seed(7722)
ObsData <- generateData(n = 10000)
write.csv(ObsData, "ObsData.csv")

# Naive approach: conditional odds ratio
naive <-glm(data = ObsData, Y ~ A + w1 + w2 + w3 + w4, family = binomial)
summary(naive)
exp(naive$coef[2])
exp(confint(naive))
```

```
## TMLE implementation by hand

# Step 1 estimation and prediction of the model for the outcome (G-computation)
gm  <- glm(Y ~ A + w1 + w2 + w3 + w4, family = binomial, data = ObsData)
# Prediction for A, A = 1 and, A = 0
QAW_0 <- predict(gm, type = "response")
Q1W_0 = predict(gm, newdata=data.frame(A = 1, ObsData [,c("w1","w2","w3","w4")]), type = "response")
Q0W_0 = predict(gm, newdata=data.frame(A = 0, ObsData [,c("w1","w2","w3","w4")]), type = "response")
# Estimated mortality risk difference
mean(Q1W_0 - Q0W_0)
# Estimated MOR
mean(Q1W_0)*(1-mean(Q0W_0))/((1-mean(Q1W_0))*mean(Q0W_0))

# Step 2 estimation and prediction of the propensity score (ps)
psm <- glm(A ~ w1 + w2 + w3 + w4, family = binomial, data = ObsData)
gW = predict(psm, type = "response")
summary(gW)

# Step 3 computation of H (clever covariates) and estimation of epsilon
H1W = ObsData$A / gW
H0W = (1-ObsData$A)  / (1 - gW)
epsilon <- coef(glm(ObsData$Y ~ -1 + H0W + H1W + offset(qlogis(QAW_0)), family = binomial))

# Step 4 Targeted estimate of the ATE and Marginal Odds Ratio
Q1W_1 <-  plogis(qlogis(Q1W_0) +  epsilon[2] / gW)
Q0W_1 <-  plogis(qlogis(Q0W_0) +  epsilon[1] / (1-gW))

    # ATE
    ATEtmle1 <-  mean(Q1W_1 - Q0W_1); ATEtmle1
    cat("\n ATEtmle1_bias:", abs(True_ATE - ATEtmle1))
    cat("\n ATEtmle1_rel_bias:",abs(True_ATE - ATEtmle1)/True_ATE,"%")

    # Marginal OR
    tmle1.MOR <- mean(Q1W_1) * (1 - mean(Q0W_1)) / ((1 - mean(Q1W_1)) * mean(Q0W_1)); tmle1.MOR

# Table to visualize the data
psi <- Q1W_1 - Q0W_1
library(DT)
df <- round(cbind(Q1W_0, Q0W_0, gW, eps1=epsilon[1], eps2=epsilon[2], psi), digits = 4)
datatable(head(df, n = nrow(df)), options = list(pageLength = 5, digits = 3))

# Step 5 statistical inference (efficient influence curve)
    # Efficient influence curve ATE
    EY1tmle<-mean(Q1W_1)
    EY0tmle<-mean(Q0W_1)
    d1 <- ((ObsData$A) * (ObsData$Y - Q1W_1)/gW) + Q1W_1 - EY1tmle
    d0 <- ((1 - ObsData$A) * (ObsData$Y - Q0W_1))/(1 - gW)  + Q0W_1 - EY0tmle
    IC <- d1 - d0
    n <- nrow(ObsData)
    varHat.IC <- var(IC) / n
    ATEtmle1CI <- c(ATEtmle1 - 1.96 * sqrt(varHat.IC), ATEtmle1 + 1.96 * sqrt(varHat.IC)); ATEtmle1;
    ATEtmle1CI
```

```
    # Efficient influence curve MOR
    ICmor_tmle <- (1 - EY0tmle) / EY0tmle / (1 - EY1tmle)^2  * d1 - EY1tmle / (1 - EY1tmle) / EY0tmle^2  *
    d0
    varHat2.IC <- var(ICmor_tmle) / n
    tmle1_ORCI <- tmle1.MOR + c(-1.96,1.96)*sqrt(varHat2.IC); tmle1.MOR; tmle1_ORCI

# Augmented inverse probability treatment weighting (AIPTW) estimator
EY1aiptw <- mean((ObsData$A) * (ObsData$Y - Q1W_0) / gW + Q1W_0)
EY0aiptw <- mean((1 - ObsData$A) * (ObsData$Y - Q0W_0) / (1 - gW) + Q0W_0)

AIPTW_ATE <- EY1aiptw - EY0aiptw; AIPTW_ATE
cat("\n AIPTW_bias:", abs(True_ATE - AIPTW_ATE))
cat("\n AIPTW_rel_bias:",abs(True_ATE - AIPTW_ATE) / True_ATE,"%")

D1 <- (ObsData$A) * (ObsData$Y - Q1W_0) / gW + Q1W_0 - EY1aiptw
D0 <- (1 - ObsData$A) * (ObsData$Y - Q0W_0) / (1 - gW) + Q0W_0 - EY0aiptw
varHat_AIPTW <- var(D1 - D0) / n

    # AIPTW ATE 95%CI
    ATEaiptw_CI <- c(AIPTW_ATE - 1.96 * sqrt(varHat_AIPTW), AIPTW_ATE + 1.96 *
    sqrt(varHat_AIPTW)); AIPTW_ATE; ATEaiptw_CI

    # AIPTW MOR 95%CI
    AIPTW_MOR <- (EY1aiptw * (1 - EY0aiptw))/((1 - EY1aiptw) * EY0aiptw);AIPTW_MOR
    ICmor_aiptw <- (1 - EY0aiptw) / EY0aiptw / (1 - EY1aiptw)^2 * D1 - EY1aiptw / (1 - EY1aiptw) /
    EY0aiptw^2 * D0
    varHat_AIPTW2 <- var(ICmor_aiptw) / n
    MORaiptw_CI <-c(AIPTW_MOR - 1.96*sqrt(varHat_AIPTW2), AIPTW_MOR +
    1.96*sqrt(varHat_AIPTW2)); AIPTW_MOR; MORaiptw_CI

# R-package tmle (base implementation includes SL.step, SL.glm and SL.glm.interaction)
library(tmle)
library(SuperLearner)
TMLE2 <- tmle(Y = ObsData$Y, A = ObsData$A, W = ObsData[,c("w1", "w2", "w3", "w4")], family =
"binomial")

#NOTE:
#Note that the tmle function default bounds the probabilities in the clever covariate denominators at 0.025.
#You can remove this bound by specifying gbound=0

ATEtmle2 <- TMLE2$estimates$ATE$psi;ATEtmle2
TMLE2$estimates$ATE$CI
MORtmle2 <- TMLE2$estimates$OR$psi;MORtmle2
TMLE2$estimates$OR$CI

cat("\n ATEtmle2_bias:", abs(True_ATE - ATEtmle2))
cat("\n ATEtmle2_Rel_bias:",abs(True_ATE - ATEtmle2) / True_ATE,"%")
```

**# R-package tmle with user-selected Super learner libraries**

```
library(tmle)
library(SuperLearner)

SL.library <- c("SL.glm","SL.step","SL.step.interaction", "SL.glm.interaction","SL.gam",
              "SL.randomForest", "SL.rpart")

TMLE3 <- tmle(Y = ObsData$Y,A = ObsData$A,W = ObsData [,c("w1", "w2", "w3", "w4")],
            family = "binomial", Q.SL.library = SL.library,g.SL.library = SL.library)

ATEtmle3 <- TMLE3$estimates$ATE$psi;ATEtmle3
TMLE3$estimates$ATE$CI
MORtmle3 <- TMLE3$estimates$OR$psi;MORtmle3
TMLE3$estimates$OR$CI

cat("\n ATEtmle3_bias:", abs(True_ATE - ATEtmle3))
cat("\n ATEtmle3_rel_bias:",abs(True_ATE - ATEtmle3) / True_ATE,"%")
```

**More complex data-generating process**

Readers interested in simulating more complex dependence structures among the covariates W1-W4 could potentially use the R-package **simcausal** (Sofrygin O, van der Laan MJ, Neugebauer R (2015). *simcausal: Simulating Longitudinal Data with Causal Inference Applications.* R package version 0.5).
See the example here below:

```
library(simcausal)
M <- DAG.empty()
M <- M +
  node("w1", # age (0/1); 1 -> high age
      distr = "rbern",
      prob = .5) +
  node("w2", # ses (1/2/3/4/5); higher age, higher probability of belonging to upper class
      distr = "rcat.b1",
      probs = {
        plogis(-3.1  + 0.05*w1); # upper middle class, 4%
        plogis(-1.25 + 0.04*w1); # middle class, 22%
        plogis(-0.05 + 0.03*w1); # lower middle  49%
        plogis(-1.65 + 0.02*w1); # working class  16%
        plogis(-2.3  + 0.01*w1)}) +
  node("w3", #comorbidities (1/2/3/4/5);
      distr = "rcat.b1",
      probs = {
        plogis(-0.8 + 0.005*w1 + 0.1*w2);
        plogis(-0.1 + 0.010*w1 + 0.12*w2);
        plogis(-1.2 + 0.015*w1 + 0.15*w2);
        plogis(-1.6 + 0.020*w1 + 0.2*w2);
        plogis(-2.5 + 0.025*w1 + 0.25*w2)}) +
  node("w4", # stage (1/2/3/4); # the higher the worse
      distr = "rcat.b1",
      probs = {
        plogis(-1 + 0.01*w1 - 0.04*w2);
        plogis(-0.2 + 0.02*w1 - 0.05*w2);
        plogis(-0.8 + 0.03*w1 - 0.055*w2);
        plogis(-2 + 0.04*w1 - 0.1*w2)}) +
```

```
   node("a", # a = 0 mono therapy; a = 1 dualtherapy
        distr = "rbern",
        prob = plogis(-1.4 + 0.05*w1 + 0.25*w3 + 0.1*exp(w4))) +
   node("y", #y = 0 -> death; y = 1 -> alive
        distr = "rbern",
        prob = plogis(-3.4 + 0.75*a - 0.1*w1 + 0.35*w2 + 0.25*w3 + 0.20*sqrt(1/w4) - 0.9*a*w2 + 1.1*a*w3))
Mset <- set.DAG(M)

# simulate observed data
Odat <- simcausal::sim(DAG = Mset, n = 10000, rndseed = 7693)

# specify the two interventions
a1 <- node("a", distr = "rbern", prob = 1)
Mset <- Mset + action("a1", nodes = a1)
a0 <- node("a", distr = "rbern", prob = 0)
Mset <- Mset + action("a0", nodes = a0)

# counterfactual data
dat <- simcausal::sim(DAG = Mset, actions = c("a1", "a0"), n = 1000000, rndseed = 7693)
head(dat[["a1"]]); head(dat[["a0"]])

# E(y) under a=1 (chemo)
Mset <- set.targetE(Mset, outcome = "y", param = "a1")
eval.target(Mset, data = dat)$res

# E(y) unter a=0 (chemo and radio)
Mset <- set.targetE(Mset, outcome = "y", param = "a0")
eval.target(Mset, data = dat)$res
# ATE (additive scale)
Mset <- set.targetE(Mset, outcome = "y", param = "a1-a0")
eval.target(Mset, data = dat)$res
# multiplicative scale
Mset <- set.targetE(Mset, outcome = "y", param = "a1/a0")
eval.target(Mset, data = dat)$res

#DAG
plotDAG(Mset, xjitter = 0.3, yjitter = 0.04,edge_attrs = list(width = 0.5, arrow.width = 0.2, arrow.size = 0.3),
vertex_attrs = list(size = 12, label.cex = 0.8))
```

## 2. STATA code for manual TMLE estimation of the ATE

```
cd "your path to the data"
import delimited ObsData.csv, clear
set more off

* Step 1: prediction model for the outcome Q0 (g-computation)
glm y a w1 w2 w3 w4, fam(binomial)
predict double QAW_0, mu
gen aa=a
replace a = 0
predict double Q0W_0, mu
replace a = 1
predict double Q1W_0, mu
replace a = aa
```

```
drop aa

* Q to logit scale
gen logQAW = log(QAW / (1 - QAW))
gen logQ1W = log(Q1W / (1 - Q1W))
gen logQ0W = log(Q0W / (1 - Q0W))

* Step 2: prediction model for the treatment g0 (IPTW)
glm a w1 w2 w3 w4, fam(binomial)
predict gw, mu
gen double H1W = a / gw
gen double H0W = (1 - a) / (1 - gw)

* Step 3: Computing the clever covariate H(A,W) and estimating the parameter (epsilon) (MLE)
glm y H1W H0W, fam(binomial) offset(logQAW) noconstant
mat a = e(b)
gen eps1 = a[1,1]
gen eps2 = a[1,2]

* Step 4: update from Q0 to Q1
gen double Q1W_1 = exp(eps1 / gw + logQ1W) / (1 + exp(eps1 / gw + logQ1W))
gen double Q0W_1 = exp(eps2 / (1 - gw) + logQ0W) / (1 + exp(eps2 / (1 - gw) + logQ0W))

* Step 5: Targeted estimate of the ATE
gen ATE = (Q1W_1 - Q0W_1)
summ ATE
global ATE = r(mean)

* Step 6: Statistical inference (efficient influence curve)
qui sum(Q1W_1)
gen EY1tmle = r(mean)
qui sum(Q0W_1)
gen EY0tmle = r(mean)

gen d1 = ((a * (y - Q1W_1)/gw)) + Q1W_1 - EY1tmle
gen d0 = ((1 - a) * (y - Q0W_1)/(1 - gw))  + Q0W_1 - EY0tmle

gen IC = d1 - d0
qui sum IC
gen varIC = r(Var) / r(N)

global LCI =  $ATE - 1.96*sqrt(varIC)
global UCI =  $ATE + 1.96*sqrt(varIC)
display "ATE:"  %05.4f  $ATE _col(15) "95%CI: " %05.4f  $LCI "," %05.4f  $UCI
```

Alternatively, one may consider exporting the data from Stata ("export delimited using "your path/yourdata.csv", replace") and then reading the data into R (read.csv("your path/yourdata.csv")) and follow the steps explained in the main manuscript.

### 3. Link to our GitHub repository for the implementation of TMLE in Stata:

The following link provides access to a developmental free testing version of TMLE implemented in Stata software.

More details and instructions for installation are provided at the following links:

https://github.com/migariane/meltmle

https://github.com/migariane/weltmle

**Example**

```
*******************************************************
* eltmle Y X Z [if] [,tmle tmlebgam]
*******************************************************
use http://www.stata-press.com/data/r14/cattaneo2.dta
describe
gen lbw = cond(bweight<2500,1,0.)
lab var lbw "Low birthweight, <2500 g"
save "your path/cattaneo2.dta", replace
cd "your path"

eltmle lbw mbsmoke mage medu prenatal mmarried, tmle
```

To replicate the results in the **box 9** from the tutorial you can type:

```
cd "your path to the data"
import delimited ObsData.csv, clear
set more off

eltmle y a w1 w2 w3 w4, tmle
```

**4. <u>R code for simulations</u> (Table 2)**

```
# Super Learner libraries
SL.library <- c("SL.glm","SL.step","SL.step.interaction","SL.glm.interaction",
                "SL.gam","SL.randomForest","SL.glmnet")

# Data generation A: dual misspecification for the model of the outcome and treatment
set.seed(7777)
generateData<- function(n){
  w1 <- rbinom(n, size=1, prob=0.5)
  w2 <- rbinom(n, size=1, prob=0.65)
  w3 <- round(runif(n, min=0, max=4), digits=0)
  w4 <- round(runif(n, min=0, max=5), digits=0)
    A <- rbinom(n, size=1, prob= plogis(-5 +  0.5*w2 + 0.25*w3 + 0.6*w4 + 0.4*w2*w4))
 # counterfactuals
  Y.1 <- rbinom(n, size=1, prob= plogis(-1 + 1 -0.1*w1 + 0.35*w2 + 0.25*w3 + 0.20*w4 + 0.15*w2*w4))
  Y.0 <- rbinom(n, size=1, prob= plogis(-1 + 0 -0.1*w1 + 0.35*w2 + 0.25*w3 + 0.20*w4 + 0.15*w2*w4))
# Observed outcome
  Y <- Y.1*A + Y.0*(1 - A)
  # return data.frame
  data.frame(w1, w2, w3, w4, A, Y, Y.1, Y.0)
}
# Data generation B: misspecification for the model of the outcome
#set.seed(7777)
#generateData<- function(n){
 # w1 <- rbinom(n, size=1, prob=0.5)
 # w2 <- rbinom(n, size=1, prob=0.65)
 # w3 <- round(runif(n, min=0, max=4), digits=0)
 # w4 <- round(runif(n, min=0, max=5), digits=0)
 #   A <- rbinom(n, size=1, prob= plogis(-5 + 0.7*w1 + 0.5*w2 + 0.25*w3 + 0.6*w4))
  # counterfactuals
 # Y.1 <- rbinom(n, size=1, prob= plogis(-1 + 1 -0.1*w1 + 0.35*w2 + 0.25*w3 + 0.15*w2*w4))
 # Y.0 <- rbinom(n, size=1, prob= plogis(-1 + 0 -0.1*w1 + 0.35*w2 + 0.25*w3 + 0.15*w2*w4))
  # Observed outcome
#  Y <- Y.1*A + Y.0*(1 - A)
  # return data.frame
```

```
#  data.frame(w1, w2, w3, w4, A, Y, Y.1, Y.0)
#}

# True ATE
ObsDataTrueATE   <- generateData(n=5000000)
True_ATE  <- mean(ObsDataTrueATE$Y.1 - ObsDataTrueATE$Y.0);True_ATE
True_EY.1 <- mean(ObsDataTrueATE$Y.1)
True_EY.0 <- mean(ObsDataTrueATE$Y.0)
True_MOR  <- (True_EY.1 * (1-True_EY.0)) / ((1-True_EY.1) * True_EY.0);True_MOR

#Simulations
library(tmle)
library(SuperLearner)
R <- 1000
#Empty vectors
naive_OR    <- rep(NA,R)
ATEtmle1   <- rep(NA,R)
MORtmle1  <- rep(NA,R)
AIPTW       <- rep(NA,R)
MOR_AIPTW <- rep(NA,R)
ATEtmle2   <- rep(NA,R)
MORtmle2  <- rep(NA,R)
ATEtmle3   <- rep(NA,R)
MORtmle3  <- rep(NA,R)

for(r in 1:R){
print(paste("This is simulation run number",r))
CancerData <- generateData(n=1000)

# ATE naive approach
naive_OR[r] <- exp(glm(data = CancerData, Y ~ A + w1 + w2 + w3 + w4, family = "binomial")$coef[2])

# TMLE implementation by hand
  # Step 1
  gm <- glm(Y ~ A + w1 + w2 + w3 + w4, family="binomial", data=CancerData)

# Prediction for A, A=1 and, A=0
  QAW <- predict(gm)
  Q1W = predict(gm, newdata=data.frame(A = 1, CancerData[,c("w1","w2","w3","w4")]))
  Q0W = predict(gm, newdata=data.frame(A = 0, CancerData[,c("w1","w2","w3","w4")]))

# Step 2 estimation of the propensity score (ps)
  psm <- glm(A ~ w1 + w2 + w3 + w4, family = binomial, data=CancerData)
  gW = predict(psm, type = "response")
  g1W = (1 / gW)
  g0W = (-1 / (1-gW))

# Step 3 computation of H and estimation of epsilon
 HAW <- (CancerData$A / gW -(1-CancerData$A) / (1 - gW))
 H1W = (1/gW)
 H0W = (-1 / (1 - gW))
 epsilon <- coef(glm(CancerData$Y ~ -1 + HAW + offset(QAW), family = "binomial"))

# Step 4 updated ATE
ATEtmle1[r] <- mean(plogis(Q1W + epsilon * H1W) - plogis(Q0W + epsilon * H0W))

# Step 5 updated MOR
  T1.EY1 <- mean(plogis(Q1W + epsilon * H1W))
  T1.EY0 <- mean(plogis(Q0W + epsilon * H0W))
MORtmle1[r] <- (T1.EY1 * (1-T1.EY0)) / ((1-T1.EY1) * T1.EY0)
```

```
# Augmented inverse probability treatment weight (AIPTW) estimator
AIPTW[r]  <- mean((HAW*(CancerData$Y - plogis(QAW)) + (plogis(Q1W)-plogis(Q0W))))
  AIPTW1  <- mean(CancerData$A * (CancerData$Y - plogis(Q1W)) / gW + plogis(Q1W) )
  AIPTW0  <- mean((1- CancerData$A) * (CancerData$Y - plogis(Q0W)) / (1-gW) + plogis(Q0W))
MOR_AIPTW[r] <- (AIPTW1 * (1- AIPTW0)) / ((1- AIPTW1) * AIPTW0)

# R-package tmle (base implementation includes SL.step, SL.glm and SL.glm.interaction)
ATE2 <- tmle(Y=CancerData$Y, A=CancerData$A,
W=CancerData[,c("w1","w2","w3","w4")], f       amily="binomial")
ATEtmle2[r] <- ATE2$estimates$ATE$psi
CORtmle2[r] <- ATE2$estimates$OR$psi

# Improved Super learner
ATE3 <- tmle(Y = CancerData$Y, A=CancerData$A, W=CancerData[,c("w1","w2","w3","w4")],
family="binomial", Q.SL.library=SL.library, g.SL.library=SL.library)
ATEtmle3[r] <- ATE3$estimates$ATE$psi
MORtmle3[r] <- ATE3$estimates$OR$psi
}

# Mean naive
mean(naive_OR)
# Mean AIPTW
mean(AIPTW)
mean(MOR_AIPTW)
# Estimate of TMLE
mean(ATEtmle1)
mean(MORtmle1)
# Estimate of TMLE + SL
mean(ATEtmle2)
mean(MORtmle2)
# Estimate of TMLE + SL2
mean(ATEtmle3)
mean(MORtmle3)
save.image("your path\results.RData")
```

## 5. Super Learner, cross-validation and ensemble learning

With TMLE we can call the R-package Super-Learner (SL). The SL combines cross-validation and ensemble learning techniques to improve model prediction and performance.[1] The SL algorithm provides a system based on V-fold cross-validation[2-4] (e.g. 10-fold) to combine multiple algorithms into an improved algorithm and returns a function that can be used for prediction in new datasets. The principal interest in calling the Super-Learner is to obtain the less-biased prediction estimates of $\overline{Q}^0(A, \mathbf{W})$ and g(A,$\mathbf{W}$).

Briefly, the SL algorithm can be described in four steps:

1) First split the data into blocks of equal size (i.e. ten blocks of 100 observations for a sample size of 1,000 units and 10-fold cross-validation) and fit each of the selected algorithms on the training set  (i.e. the 9 grey blocks in Supplementary Figure 2).

2) Then, predict the estimated probabilities of the outcome (Y) using the validation set (i.e. the black blocks in Supplementary Figure 2) for each algorithm, based on the corresponding training set;

3) Repeat steps 1 and 2 for each of the ten blocks (R2-R10 in Supplementary Figure 1).

4) Afterwards, the SL estimates the cross-validation risk for each algorithm, e.g. the sum of the squared differences between the predicted and actual values of Y.

5) Finally, the SL chooses the weighted combination of algorithms that minimises the cross-validated risk (e.g.mean squared error). This combination is linear, the weights are non-negative and sum up to one.[1,3] For a binary outcome, this relates to estimate a non-negative least squares where the ensemble of cross-validated predictions, for each algorithm (Z) are used as independent variables to predict the outcome (Y).

**References**


1. Breiman L. *Stacked regressions. Machine learning. 1996;24(1):49--64.*
2. Efron B, Efron B. *The jackknife, the bootstrap and other resampling plans.* Vol 38: SIAM; 1982.
3. van der Laan MJ, Polley EC, Hubbard AE. Super learner. *Stat Appl Genet Mol Biol.* 2007;6:Article25.
4. Gruber S, van der Laan M. tmle: An R Package for Targeted Maximum Likelihood Estimation. *U.C. Berkeley Division of Biostatistics Working Paper Series.* 2011.
